# Developmental Trajectories of Joint Attention and Behavioral Request in Infancy: A Longitudinal Study from 8 to 18 Months

**DOI:** 10.3390/children13070954

**Published:** 2026-07-20

**Authors:** Maite Montagut-Asunción, Ana D’Ocon-Giménez, Gemma Pastor-Cerezuela

**Affiliations:** Department of Basic Psychology, University of Valencia, 46010 Valencia, Spain; ana.ocon@uv.es (A.D.-G.); gemma.pastor@uv.es (G.P.-C.)

**Keywords:** joint attention, behavioral request, early social communication, infancy, developmental trajectories, developmental risk, neurodevelopmental disorders

## Abstract

**Highlights:**

**What are the main findings?**

- Joint attention and behavioral request follow distinct developmental patterns across infancy, reflecting differences in their communicative functions (declarative vs. imperative). Within joint attention, early low-level behaviors remain relatively stable over time, whereas high-level behaviors show significant increases, particularly between 12 and 18 months.
- Behavioral request behaviors also exhibit differentiated developmental trajectories, with low-level behaviors peaking at 12 months and declining thereafter, while high-level behaviors increase progressively from 12 to 18 months.

**What are the implications of the main findings?**

- Understanding normative developmental trajectories of joint attention and behavioral request can support the early identification of atypical developmental patterns associated with neurodevelopmental disorders.
- Assessing multiple dimensions of early social communication may enhance the sensitivity of early screening and intervention strategies.

**Abstract:**

**Background/Objectives:** Joint attention and behavioral request are foundational component of early social communication and play a crucial role in later socio-cognitive development. This study aimed to examine the developmental trajectories of joint attention and behavioral request behaviors across infancy. **Methods:** A longitudinal design was employed with a birth cohort of 55 infants from Valencia, Spain, assessed at 8, 12, and 18 months of age. Joint attention and behavioral request behaviors were evaluated using the Early Social Communication Scales (ESCS). Linear mixed-effects models (LMMs) were fitted to examine developmental changes over time. **Results:** The findings revealed differentiated developmental trajectories depending on the level of behavioral complexity. Low-level Initiating Joint Attention remained stable across time, whereas high-level Initiating and Responding to Joint Attention behaviors increased significantly with age. Similarly, behavioral request behaviors showed a shift from early low-level forms to more complex and intentional behaviors over time. Significant developmental changes were observed for both joint attention and behavioral request across ages. **Conclusions:** Joint attention and behavioral request follow a hierarchical developmental pattern during infancy, characterized by early stability in basic behaviors and later emergence of more complex communicative forms. These findings contribute to a better understanding of typical development and highlight the relevance of early social communication behaviors as potential markers of developmental risk.

## 1. Introduction

**Figure 1:**
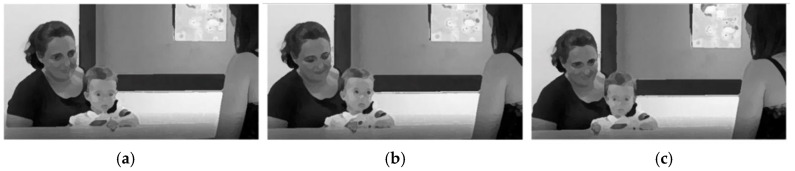
Example of gaze alternation in an infant during a joint attention episode (**a**–**c**). The examiner, on the right side of the images, faces the infant (with her back to the camera), while the infant alternates their gaze between the object and the examiner. The mother is present but does not intervene, keeping the infant secure during the assessment. Note: As facial expressions are essential for interpreting the behaviors of interest, the images could not be pixelated or blurred; instead, visual filters were applied to reduce the likelihood of participant identification.

Joint attention is the ability to coordinate attention with another person in relation to an object or event [1,2,3]. It is a clear indicator of understanding the perspectives of others [4] and plays an important role in the emergence of symbolic thinking and in the development of language [2,5]. This skill mainly has a social function—to share motivations and interests with others (proto-declarative gestures or declarative joint attention) [6,7]—but it can also have an instrumental role when used to obtain an object or achieve a goal (proto-imperative gestures or imperative joint attention) [8]. A typical example of joint attention can be observed when a baby looks intently at a string toy that his mother is holding. Then the mother activates the toy while the baby watches it carefully. He is smiling while alternating his gaze between the toy and his mother’s face, showing interest in the shared game, and evidencing that he understands that there is a common interest. This pre-linguistic communicative skill typically begins to emerge around 10–12 months of age [9] (Figure 1).

**Figure 2:**
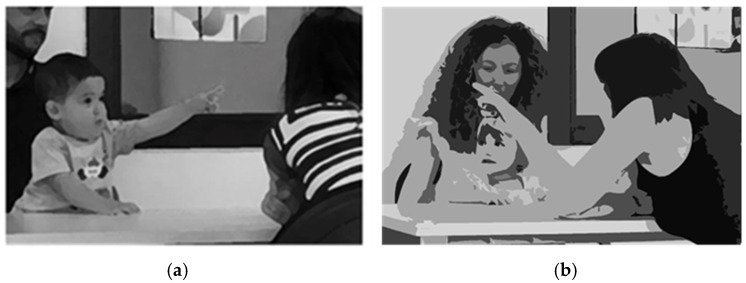
(**a**) Example of Initiating Joint Attention (IJA): The infant points to a picture on the wall while maintaining eye contact with the examiner (on the right side of the image). The father is present but does not intervene. (**b**) Example of Responding to Joint Attention (RJA): The infant looks at a picture on the wall following the examiner’s pointing gesture. The mother is present but does not intervene.

Commonly, the term joint attention is used to refer to the declarative purpose of this communicative behavior, whereas the term behavioral request is usually used to refer to its imperative function [2,10,11,12]. It is also important to distinguish between Initiating Joint Attention (IJA) and Responding to Joint Attention (RJA), as these two dimensions reflect different processes during child development [1,13], rely on different mechanisms, and engage distinct neural systems [14,15]. Initiating Joint Attention (IJA) occurs when the infant spontaneously initiates an episode of shared attention with another person. In contrast, Responding to Joint Attention (RJA) occurs when the infant follows another person’s gaze or gesture to share attention toward an object or event (Figure 2). The same distinction can be applied to behavioral requests. When a behavioral request episode is initiated by a child, it is referred to as Initiating Behavioral Request (IBR). For example, the child may alternate their gaze between an adult and a mechanical toy to prompt the adult to reactivate it. In contrast, when the adult initiates the episode—for instance, by asking the child to give or show an object—it is referred to as responding to behavioral request (RBR) [1].

### 1.1. Joint Attention as a Precursor to Other Socio-Communicative Milestones

The role of joint attention in socio-communicative development can be understood within the Parallel and Distributed Processing Model (PDPM) [10,11,14]. This framework conceptualizes joint attention as a parallel and distributed process, involving the simultaneous processing of information about one’s own attention and that of others, and relying on widespread neural networks rather than isolated brain regions. From this perspective, it emerges from increasingly efficient and coordinated processing mechanisms, within a developmental continuum that extends from early perceptual and attentional processes to higher-order socio-cognitive abilities. Accordingly, the development of joint attention is grounded in earlier processes such as gaze following and joint perception, and precedes the consolidation of higher-order abilities including symbolic thinking, language, mentalization, theory of mind, and broader social cognition [10,14,16]. Extending this perspective, recent naturalistic studies suggest that coordinated attention may emerge through multiple sensorimotor pathways, including gaze following, manual actions, and object manipulation, highlighting the multimodal nature of early social communication [17]. Recent longitudinal research has shown that individual differences in early social communication trajectories are associated with later developmental outcomes, including language abilities and neurodevelopmental trajectories [18,19,20,21].

Impairments in joint attention are considered a core feature of the social communication difficulties observed in autism spectrum disorder (ASD) [22,23,24]. Similarly, difficulties in joint attention and gesture use have also been reported in other neurodevelopmental disorders and developmental risk conditions [25]. Although ASD is a highly heterogeneous neurodevelopmental condition, with social communication features varying considerably in their severity and clinical presentation across individuals [26], early social communication difficulties remain a defining characteristic. Longitudinal studies have shown that infants later diagnosed with ASD already exhibit fewer social-communication behaviors by 9 months of age [18]. Similarly, reduced gesture use, particularly in joint attention and gesture integration, has been identified as an early behavioral indicator of ASD, with differences emerging during the second year of life [27]. These early alterations in non-verbal social communication are particularly relevant, as they emerge during the first stages of development and may serve as early behavioral markers of developmental risk.

### 1.2. Behavioral Request as an Early Form of Intentional Communication

Although joint attention has received greater attention in the early development literature, increasing attention has also been paid to other communicative skills, such as gesture production and behavioral request, and their integration into early social communication as early indicators of developmental risk [27].

Behavioral request refers to the infant’s ability to intentionally regulate another person’s behavior to obtain a desired object, action, or assistance. It represents an important milestone in the emergence of intentional communication and constitutes a fundamental foundation for the development of spoken language. It reflects the infant’s understanding that other people can be used as social partners to achieve communicative goals [8]. Behavioral request skills typically emerge during the second half of the first year of life, become well established by around 12 months, and are increasingly integrated with speech during the second year, before gradually giving way to verbal communication as language develops [27]. Longitudinal studies have shown that early communicative gestures, including imperative gestures, are associated with subsequent language development and broader communicative competence [28,29]. Delays or atypical trajectories in these early communicative behaviors have been associated with a range of neurodevelopmental disorders, highlighting their value for the early identification of children at risk of developmental difficulties [27].

Thus, joint attention and behavioral request, together with other communicative behaviors, form part of a broader system of early social communication skills that supports later socio-cognitive development. Given the importance of these abilities for both typical development and the identification of neurodevelopmental disorders, such as ASD, Developmental Coordination Disorder (DCD), and Specific Language Impairment (SLI), examining how they emerge and evolve during infancy is particularly relevant, as the assessment of early social communication may improve the sensitivity of developmental screening. Early diagnosis is associated with better long-term outcomes, as timely intervention can improve cognitive, social, and adaptive functioning in children with neurodevelopmental impairments [30,31,32].

The aim of the present study was to analyze the developmental trajectories of joint attention and behavioral request across 8, 12, and 18 months in a birth cohort of children born in the province of Valencia, Spain.

## 2. Materials and Methods

### 2.1. Participants

The sample consisted of 55 infants born in the province of Valencia, Spain (24 boys, 43.6%, and 31 girls, 56.4%) and their families. Of the participating infants, 38 (69.1%) were born at term (>36 weeks of gestation) and 17 (30.9%) were born preterm (<37 weeks of gestation). In the case of preterm infants, corrected age was used for all age-based criteria and analyses. Preterm infants were recruited through the Neonatology Unit at Hospital La Fe and underwent routine follow-up by the clinical team after discharge. According to their routine clinical follow-up, none presented evidence of developmental impairment or severe medical complications, and all met the predefined inclusion and exclusion criteria. Therefore, they were considered typically developing for the purposes of the present study.

Among the sample, a total of 47 infants (85.5%) were singletons, whereas 8 (14.5%) were twins, corresponding to four twin pairs included in the study. Among the twins, 7 (87.5%) were girls and 1 (12.5%) was a boy. Overall, 98.2% of births (*n* = 54) occurred without peri- or postnatal complications. In only one case, the mother reported neonatal hypotonia, the need for oxygen resuscitation, and an Apgar score of 6 at birth. Nevertheless, according to routine clinical follow-up by the hospital, no developmental impairment or relevant medical complications were identified during the assessment period, and the infant met all predefined inclusion and exclusion criteria.

The study followed a prospective longitudinal birth cohort design with three assessment points at 8, 12, and 18 months of age. Participants were recruited between 2017 and 2019 using a convenience sampling approach through collaborations with primary healthcare centers in the city of Valencia and the Neonatology Unit at Hospital La Fe, where healthcare professionals informed eligible families about the study and invited them to participate. A total of 52 infants were assessed at 8 months, 44 at 12 months, and 30 at 18 months. To examine the possibility of attrition bias, baseline socio-demographic characteristics and 8-month social communication measures were compared between participants who completed all three assessments and those who did not. No significant group differences were observed in baseline joint attention or behavioral request measures, sex, prematurity, twin status, birth weight, gestational age, number of siblings, or father age (all *p*s > 0.05). Maternal age was the only variable that differed significantly between groups, with mothers of infants who completed all three assessments being older than those of non-completers (*p* = 0.012). Overall, these findings suggest that attrition was largely unrelated to the infants’ baseline socio-demographic and developmental characteristics and was therefore unlikely to have introduced substantial attrition bias.

Participants were recruited through collaborations with professionals from health centers and hospitals in Valencia. Health professionals informed families about the study and invited them to participate. Families who expressed interest were contacted by the research team and received detailed information about the objectives and procedures of the study. Participation was voluntary, and parents provided informed consent prior to their child’s inclusion in the study. The participating families were predominantly nuclear (90.9%) and resided mainly in urban (67.3%) or residential (23.6%) areas. The largest proportion of families reported an annual household income between €24,000 and €35,999 (34.5%), followed by €12,000–23,999 (29.1%) and €36,000–50,000 (21.8%). Parents were, on average, in their mid-thirties (mothers: *M* = 34.8 years, *SD* = 4.76; fathers: *M* = 36.9 years, *SD* = 5.07). A substantial proportion of parents had completed higher education, including 63.6% of mothers and 50.9% of fathers.

#### Inclusion and Exclusion Criteria

Infants were eligible to participate if they met the following criteria: (1) Being 8 months of age at the time of recruitment (corrected age for preterm infants); (2) for full-term infants, a gestational age ≥ 37 weeks and birth weight appropriate for gestational age (AGA); (3) for preterm infants, gestational age < 37 weeks and birth weight appropriate for gestational age (AGA); (4) parental consent to participate in the assessments and for the use of the collected data; (5) at least one parent or caregiver able to understand Spanish or Valencian.

Infants were excluded if they presented: (1) any medical condition associated with an increased risk of neurodevelopmental disorders; (2) severe medical complications related to prematurity that could imply a high risk of severe cognitive, motor, or sensory impairment; (3) the presence of a metabolic, genetic, or neurodevelopmental disorder.

### 2.2. Ethics Statement

The study was approved by the Research Ethics Committee of the Hospital Universitari i Politècnic La Fe (Valencia, Spain) (approval date: 9 May 2017, protocol number 2017/0167). All procedures were conducted in accordance with the ethical standards of the Declaration of Helsinki. Written informed consent was obtained from the parents or legal guardians prior to the infants’ participation.

### 2.3. Instrument and Variables: Early Social Communication Scales (ESCS)

Early social communication skills—including joint attention and behavioral request—were assessed using the Early Social Communication Scales (ESCS; University of Miami, Miami, FL, USA) [33], a semi-structured observational instrument designed to evaluate nonverbal social communication in infants and young children through structured interactions with an examiner involving toys and social games. The assessment sessions are video-recorded and subsequently coded by one or more trained observers to identify and quantify the targeted behaviors. It has been extensively employed in developmental and autism research [34,35] and has demonstrated predictive validity for later language and socio-communicative outcomes [1,36]. The administration of the ESCS lasts approximately 30–40 min, depending on the infant’s age, level of engagement, and behavioral state.

The ESCS has demonstrated good inter-rater reliability across studies [12]. In the present study, inter-rater reliability was evaluated by randomly selecting 54.33% of the observations, which were independently coded pairwise across different combinations of coders from the research team. Four trained coders were responsible for the coding. They first completed a training phase, followed by a pilot coding phase, to ensure a consistent understanding and application of the coding criteria. Following completion of the training and pilot coding phases, formal coding was conducted. Inter-rater reliability was assessed for each ESCS dimension using intraclass correlation coefficients (ICCs). The mean inter-rater reliability values were 0.768 at 8 months, 0.814 at 12 months, and 0.814 at 18 months, indicating adequate to high agreement across assessment points. Intraclass correlation coefficients (ICCs) were not calculated for Initiating Behavioral Request—high level (IBRH) and Responding to Behavioral Request (RBR) at 8 months because all participants obtained a score of zero for these variables. Consequently, there was no between-subject variance, making ICC estimation mathematically impossible.

The ESCS differentiates between declarative communicative behaviors (joint attention) and imperative communicative behaviors (behavioral request), and distinguishes between initiating behaviors when the child initiates the communicative episode, and responding behaviors when the child responds to the adult’s cue. Moreover, it distinguishes between low-level behaviors involving basic communicative acts such as eye contact and gaze alternation, and high-level behaviors involving more advanced, intentional gestures such as pointing, showing, or giving objects. Initiating Joint Attention—low level (IJAL) is coded when the child alternates their gaze between an object and the adult, indicating an attempt to share attention. Initiating Joint Attention—high level (IJAH) is coded when the child uses more explicit communicative behaviors, such as pointing, showing, or giving objects to direct the adult’s attention. Responding to Joint Attention—proximal (RJAP) is coded when the child follows the adult’s gaze or pointing gesture towards a nearby object (e.g., a book), whereas Responding to Joint Attention—distal (RJAD) is coded when the child follows attentional cues directed towards a distant object (e.g., a picture on the wall). Initiating Behavioral Request—low level (IBRL) is coded when the child alternates their gaze between the adult and a mechanical object that is inactive, indicating an attempt to request the object or to request the activation of the object. Initiating Behavioral Request—high level (IBRH) is coded when the child employs requesting strategies such as pointing or vocalizing. Finally, Responding to Behavioral Request (RBR) is coded when the child appropriately complies with the adult’s request (e.g., giving or showing an object).

### 2.4. Analysis

Descriptive statistics (means and standard deviations) were calculated for all variables for joint attention (IJAL, IJAH, RJAP, and RJAD) and behavioral request (IBRL, IBRH, and RBR). To examine developmental changes across the three assessment points (8, 12, and 18 months), linear mixed-effects models (LMMs) were fitted using the following model: Outcome ~ Assessment point + (1|Participant), where assessment point (8, 12, and 18 months) was treated as a fixed effect and participant was included as a random intercept. Separate models were fitted for each joint attention and behavioral request. LMMs were selected because they are well suited for the analysis of longitudinal data with repeated measurements, accommodate incomplete follow-up data under the missing-at-random assumption, and account for within-participant correlations. Model assumptions were assessed by visual inspection of residual diagnostic plots, including Q–Q plots, residual histograms, and residuals-versus-predicted plots. The residuals showed no substantial deviations from normality or homoscedasticity, supporting the adequacy of the fitted models. Minor departures from these assumptions were observed for the IBRH and RBR models, primarily reflecting the discrete nature of these variables and the marked floor effects at 8 months, when these behaviors are not yet typically observed and most participants consequently obtained zero scores. Model convergence was achieved in every case. All analyses were performed using IBM SPSS Statistics (Version 26.0.; Armonk, NY, USA: IBM Corp.) and jamovi (Version 2.7.37; the jamovi project, Sydney, Australia).

## 3. Results

The results are presented separately for joint attention and behavioral request to facilitate interpretation and maintain consistency with the previous literature.

### 3.1. Joint Attention

Table 1 presents the descriptive statistics of the joint attention variables across the three assessment points (8, 12, and 18 months).

Initiating Joint Attention behaviors showed different developmental patterns depending on their level of complexity. Low-level Initiating Joint Attention (IJAL) behaviors remained relatively stable across the three time points, whereas high-level (IJAH) behaviors increased notably with age. Similarly, Responding to Joint Attention behaviors showed clear developmental gains. Both proximal Responding to Joint Attention (RJAP) and distal Responding to Joint Attention (RJAD) increased substantially between 8 and 18 months, indicating a progressive improvement in children’s ability to follow the adult’s attentional cues.

**Figure 3:**
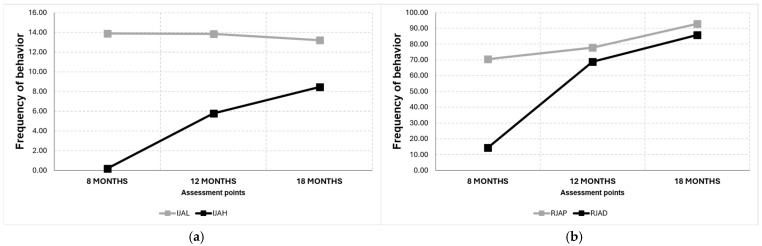
(**a**) Developmental trajectories of IJA across assessment points. (**b**) Developmental trajectories of RJA across assessment points.

Figure 3 illustrates the developmental trajectories of joint attention across assessment points.

To examine developmental changes across assessment points linear mixed-effects models (LMMs) were fitted separately for each joint attention variable. Assessment point was included as a fixed effect and participant as a random intercept. The LMM analyses revealed distinct developmental trajectories across the four joint attention behaviors. No significant effect of assessment point was observed for Initiating Joint Attention—low level (IJAL) (*F*(2, 77.6) = 0.363, *p* = 0.697). In contrast, significant effects of assessment point were found for Initiating Joint Attention—high level (IJAH) (*F*(2, 86.2) = 44.10, *p* < 0.001), Responding to Joint Attention—proximal (RJAP) (*F*(2, 73.8) = 8.05, *p* < 0.001), and Responding to Joint Attention—distal (RJAD) (*F*(2, 85.5) = 152.00, *p* < 0.001). Parameter estimates indicated significant increases from 8 to 18 months for IJAH, RJAP and RJAD, whereas IJAL remained stable across assessment points (see Table 2 for detailed statistics).

### 3.2. Behavioral Request

Table 3 presents the descriptive statistics for behavioral request variables across the three assessment points (8, 12, and 18 months).

Low-level Initiating Behavioral Request (IBRL) behaviors increased from 8 to 12 months and decreased slightly at 18 months, whereas high-level (IBRH) behaviors showed a progressive increase across the assessment points. Responding to Behavioral Request (RBR) behaviors also increased markedly over time, reflecting improvements in children’s ability to respond to the adult’s requests.

**Figure 4:**
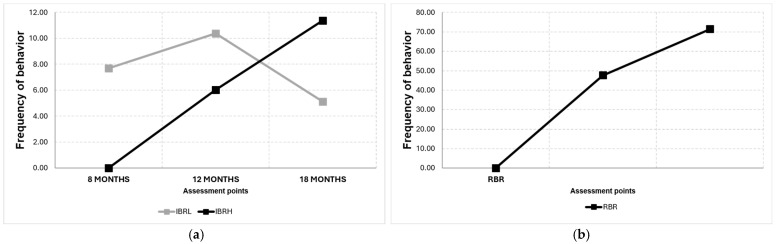
(**a**) Developmental trajectories of IBR across assessment points. (**b**) Developmental trajectories of RBR across assessment points.

Figure 4 illustrates the developmental trajectories of behavioral request across assessment points.

To examine developmental changes across assessment points, linear mixed-effects models (LMMs) were fitted separately for each behavioral request variable. Assessment point was included as a fixed effect and participant as a random intercept. Significant effects of assessment point were observed for all behavioral request variables. Specifically, assessment point significantly affected IBRL (*F*(2, 82.7) = 11.40, *p* < 0.001), IBRH (*F*(2, 87.6) = 49.70, *p* < 0.001), and RBR (*F*(2, 90.6) = 98.00, *p* < 0.001). Parameter estimates showed that IBRL increased between 8 and 12 months (Estimate = 1.88, *p* = 0.021) and subsequently decreased at 18 months (Estimate = −2.55, *p* = 0.006), whereas IBRH and RBR exhibited progressive increases across infancy (see Table 4 for detailed statistics).

## 4. Discussion

The present study aimed to examine the developmental trajectory of joint attention across three key time points (8, 12, and 18 months) in a birth cohort of children born in the province of Valencia, Spain. Overall, the findings suggest that joint attention and behavioral request follows a differentiated developmental pattern, with early-emerging behaviors remaining relatively stable and more complex forms showing a progressive increase across the first 18 months of life. This pattern is consistent with recent models emphasizing developmental trajectories and individual differences in joint attention and social cognition [11].

The results revealed differentiated developmental trajectories for joint attention and behavioral request, depending on the level of complexity and communicative function. First, lower-level joint attention (IJAL) showed a stable and high pattern across the three time points, indicating that gaze alternation is an early-emerging communicative behavior that stabilizes quickly. This finding is consistent with previous research showing that gaze alternation is observable as early as 8–9 months of age [34,37]. The slight, non-significant decrease observed between 12 and 18 months may reflect a developmental shift from simpler to more complex communicative strategies, in line with studies reporting a decrease in lower-level IJA behaviors alongside an increase in higher-level ones during this period [1,34,35,38,39,40]. Second, responding to proximal joint attention (RJAP) showed relatively high scores at early ages and a significant increase between 12 and 18 months, suggesting both early emergence and later consolidation. This pattern aligns with evidence indicating that infants are able to respond to proximal joint attention cues around 9–10 months, with further development in their ability to manage and use these cues over time [2,36]. Additionally, the distinction between proximal and distal responding is supported by previous findings showing that responses to distant objects (RJAD) typically emerge later, around 14–15 months [2].

Finally, higher-level joint attention variables (IJAH, RJAD) showed a clear increasing trajectory, with near-zero scores at 8 months and marked growth from 12 months on-wards. This suggests that more complex, gesture-based communicative behaviors emerge after the first year of life and continue to develop during the second year. These findings are consistent with prior research indicating that higher-level triadic communication behaviors, such as pointing and showing, typically emerge around 12 months and reflect increasing socio-cognitive sophistication [10,41,42]. The continued increase between 12 and 18 months may reflect the consolidation and mastery of these more advanced communicative skills [36,42].

Similarly, behavioral request variables followed a parallel pattern to joint attention. Lower-level behaviors (IBRL) showed an early presence and a slight decline over time, whereas higher-level behaviors (IBRH, RBR) showed a progressive increase. This pattern supports the idea that early communicative strategies are gradually replaced or complemented by more complex and intentional behaviors, as suggested in the previous literature [1,10,35,41].

Notably, a slightly decline between 12 and 18 months was found in lower-level behaviors of request (IBRL), which may reflect a developmental shift whereby simpler communicative strategies are progressively replaced by more complex and intentional forms of communication. This pattern is consistent with previous research suggesting that early communicative behaviors are reorganized as children acquire more advanced triadic skills [35]. In line with this, higher-level behaviors such as IBRH typically emerge after the first year of life and continue to increase during the second year, reflecting growing socio-cognitive and communicative sophistication [1,10,41]. Furthermore, the continued in-crease in IBRH observed between 12 and 18 months may indicate the consolidation and mastery of these higher-level requesting behaviors [10,36]. Taken together, these findings suggest that early social communication behaviors follow a hierarchical and progressive developmental pattern, in which lower-level behaviors (e.g., gaze alternation) emerge early and remain relatively stable, while higher-level behaviors (e.g., pointing, showing, and responding to distal cues) emerge later and show a marked increase only after the first year of life. This pattern reflects a developmental shift from basic attentional coordination to more complex, intentional forms of communication. Importantly, these results highlight that different components of joint attention and behavioral request do not develop uniformly, but rather follow distinct trajectories depending on their cognitive and communicative demands. These findings further support the view that early social communication is not a unitary construct but comprises multiple communicative functions that follow distinct developmental trajectories, consistent with recent latent trajectory analyses of early social communication development [19].

It is also important to acknowledge the inter-individual variability observed across several measures of early social communication, which highlights that, although clear developmental trends emerged at the group level, individual infants may follow different developmental trajectories while remaining within the range of typical development.

From a theoretical perspective, these findings support developmental models that conceptualize early social communication skills, especially joint attention, as a foundational mechanism underlying later socio-cognitive abilities, including language and theory of mind [1,10]. The differentiation observed between initiating and responding behaviors, as well as between proximal and distal forms, reinforces the view that early social communication is a multifaceted construct comprising distinct but interrelated processes.

From a clinical standpoint, these results have important implications for the early identification of infants at risk for autism and other developmental disabilities. Given that impairments in joint attention are considered a core feature of ASD [21,22,23], understanding the typical developmental trajectories of these behaviors is essential for identifying atypical patterns, which may serve as informative markers of developmental risk [18,43]. Moreover, the parallel developmental patterns observed between joint attention and behavioral request further support the notion that evaluating multiple dimensions of early communication may provide a more comprehensive and sensitive approach to early identification.

Despite these contributions, several limitations should be acknowledged. First, the sample size decreased across assessment points, which may have limited the statistical power of some analyses and the generalizability of the findings. Second, the study focused on a typically developing population from a specific geographical context, which may restrict the extent to which the results can be generalized to other populations. Third, although the ESCS provides a well-established observational framework, it captures behaviors within a semi-structured context, which may not fully reflect children’s spontaneous communication in naturalistic settings. In addition, the variables derived from the ESCS measurements primarily reflect frequency of occurrence of early social-communicative behaviors. Consequently, they may not fully capture qualitative aspects of social communication, such as the coordination of gaze, gestures, and vocalizations or the increasing complexity of communicative bids across development. Recent evidence suggests that these qualitative characteristics may provide additional information about early social communication development beyond behavioral frequency alone [44].

Future studies could complement frequency-based observational measures with indices of communicative quality, multimodal coordination, and interactional complexity, replicate these findings in larger and more diverse samples, including populations at risk for ASD and other neurodevelopmental disorders, and integrate observational assessments with complementary methodologies, such as parent-report instruments or eye-tracking techniques. Together, these approaches would provide a more comprehensive understanding of early communicative development. Longitudinal designs extending beyond 18 months would also be valuable to better understand how early social communication relates to later language and socio-cognitive outcomes.

## 5. Conclusions

The findings of this study provide a detailed characterization of the developmental trajectories of joint attention and behavioral request during early infancy. Joint attention showed stable low-level behaviors and increasing higher-level and responding forms over time. In contrast, behavioral request followed a more variable pattern, with early low-level behaviors increasing and later decreasing, and higher-level behaviors progressively increasing. Overall, these results contribute to a more nuanced understanding of early socio-communicative development and highlight the importance of considering the distinct developmental trajectories of different communicative functions. Such findings may inform the early identification of atypical developmental patterns and improve early screening approaches for neurodevelopmental disorders, particularly ASD, as early behavioral markers can now be identified within the first year of life [18,24].

## Figures and Tables

**Table 1:** Descriptive statistics of joint attention variables across assessment points.

Variable	8 Months *M* (*SD*)	12 Months *M* (*SD*)	18 Months *M* (*SD*)
IJAL	13.86 (8.64)	13.82 (6.77)	13.21 (8.26)
IJAH	0.18 (0.61)	5.75 (5.18)	8.46 (5.41)
RJAP	70.46 (27.04)	77.81 (28.82)	92.80 (20.03)
RJAD	14.29 (19.46)	68.90 (27.09)	85.71 (21.44)

Note. *M* = mean; *SD* = standard deviation. IJAL = Initiating Joint Attention (low level); IJAH = Initiating Joint Attention (high level); RJAP = Responding to Joint Attention (proximal); RJAD = Responding to Joint Attention (distal).

**Table 2:** Linear mixed-effects model results for joint attention variables across assessment points.

Variable	*F*(df1, df2)	*p*	12 vs. 8 (Estimate)	*p*	18 vs. 8 (Estimate)	*p*
IJAL	0.363 (2, 77.6)	0.697	0.39	0.775	−0.94	0.543
IJAH	44.10 (2, 86.2)	<0.001	5.36	<0.001	8.52	<0.001
RJAP	8.05 (2, 73.8)	<0.001	7.11	0.127	21.05	<0.001
RJAD	152.00 (2, 85.5)	<0.001	55.30	<0.001	74.60	<0.001

Note. *F* = *F* statistic for the overall effect of assessment point; Estimate = estimated difference relative to the 8-month assessment (reference category); df = denominator degrees of freedom estimated using the Satterthwaite approximation.

**Table 3:** Descriptive statistics of behavioral request variables across assessment points.

Variable	8 Months *M* (*SD*)	12 Months *M* (*SD*)	18 Months *M* (*SD*)
IBRL	7.68 (3.79)	10.39 (4.48)	5.11 (3.72)
IBRH	0.00 (0.00)	6.04 (4.66)	11.36 (7.54)
RBR	0.00 (0.00)	47.62 (33.57)	71.37 (27.88)

Note. *M* = mean; *SD* = standard deviation. IBRL = Initiating Behavioral Request (low level); IBRH = Initiating Behavioral Request (high level); RBR = Responding to Behavioral Request.

**Table 4:** Linear mixed-effects model results for behavioral request variables across assessment points.

Variable	*F*(df1, df2)	*p*	12 vs. 8 (Estimate)	*p*	18 vs. 8 (Estimate)	*p*
IBRL	11.40 (2, 82.7)	<0.001	1.88	0.021	−2.55	0.006
IBRH	49.70 (2, 87.6)	<0.001	7.58	<0.001	11.71	<0.001
RBR	98.00 (2, 90.6)	<0.001	47.60	<0.001	70.40	<0.001

Note. *F* = *F* statistic for the overall effect of assessment point; Estimate = estimated difference relative to the 8-month assessment (reference category); df = denominator degrees of freedom estimated using the Satterthwaite approximation.

## Data Availability

The data presented in this study are available on request from the corresponding author due to ethical and privacy restrictions, as they contain information from minor participants.

## Abbreviations

The following abbreviations are used in this manuscript:

AGA	Appropriate for Gestational Age
ASD	Autism Spectrum Disorder
DCD	Developmental Coordination Disorder
ESCS	Early Social Communication Scales
IBR	Initiating Behavioral Request
IBRH	Initiating Behavioral Request—High Level
IBRL	Initiating Behavioral Request—Low Level
ICC	Intraclass Correlation Coefficient
IJA	Initiating Joint Attention
IJAH	Initiating Joint Attention—High Level
IJAL	Initiating Joint Attention—Low Level
LMM	Linear Mixed-Effects model
RBR	Responding to Behavioral Request
RJA	Responding to Joint Attention
RJAD	Responding to Joint Attention—Distal
RJAP	Responding to Joint Attention—Proximal
SLI	Specific Language Impairment
